# A lesson learned about predatory journals and their difference from peer-reviewed open-access publishing

**DOI:** 10.1016/j.ijwd.2016.08.004

**Published:** 2016-10-13

**Authors:** Dedee F. Murrell

**Affiliations:** Co-Editor, International Journal of Women’s Dermatology; Professor and Chair, Dept of Dermatology, St George Hospital, University of NSW, Sydney, Australia

Ever since the Women’s Dermatologic Society (WDS) Board of Directors voted in March of 2014 to establish a peer-reviewed journal, our leadership has realized how the advent of predatory publishing has affected the establishment of a legitimate medical publication. For example, in 2014, there were already approximately 40 different dermatologic journals but none with a specific focus on women’s skin problems and career issues of female dermatology trainees. In fact, there are no specific journal publications dealing with careers for women in medicine. The WDS is the third largest dermatologic society in the United States with approximately 10% male membership holders and a growing proportion of international members. Leaders in academic dermatology, both female and male, make a significant difference in terms of the main goals of the WDS: mentorship and leadership development. The two WDS co-editors in 2014 approached a number of respected publishers to explore options to publish the journal. Not one of the medical publishers offered the WDS the possibility of a subscription-based publication, stating that with the proliferation of predatory open-access publications, most libraries were not willing to add on additional charges for new journals. Hence, the only way forward was an open access, peer-reviewed model. For many of us within the WDS, this has been hard to accept because we have matured in our careers with journals that have no publication charges and are generally accessible free-of-charge through the university or hospital we are affiliated with, or by society subscription.

In addition, many dermatologists and even trainees have become accustomed to receiving invitations almost on a daily basis from non-medical journal editors representing various “predatory journals” and asking us to write articles for them. Some of these journals have titles that are very similar to those of legitimate dermatology journals associated with prestigious dermatologic societies, such as the British Association of Dermatology’s two journals. In fact, these predatory journals have invited dermatologists to join their editorial boards as an honorary activity, so the journals appear to be legitimate on the basis of who is associated with them. I had the personal experience of being invited to submit a case report to one such journal, and after seeing the names of several respected colleagues serving on their editorial board, I believed it to be a legitimate publication. Therefore, I decided to submit an interesting case report for publication. Yet, within a couple of hours of submitting the case report, I received an email from the non-medical editor, stating it had been reviewed and was accepted for publication with no revisions. This is very unusual for any journal submission. Subsequently, what I thought was a $50 submission fee for the article turned into a $3,000 charge to my credit card! I tried to contact the journal for an explanation, and discovered that their ‘head office’ in New York was a P.O. box with an answering machine containing an outgoing message in broken English. I contacted my colleagues on the editorial board to see if they were aware that this journal was using their good names in this way and, of course, they were not. Several of them immediately resigned from the editorial board of that journal and insisted that I be refunded this fee, which was hidden in small print at the time the article was submitted.

Yet, there are some excellent journals, which are now open-access and charge publication fees, such as the Public Library of Science (PLOS) One journals. I recall in the old days, pre-internet, that we had to pay a lot to get multiple copies of high quality printed images posted in black and white. Some people would even pay typists and extra fees for the images to be printed in color in the journals. These days, we take for granted that we can electronically upload submissions for free and have forgotten that there were always associated fees, which we or our departments absorbed. To be able to give copies of our articles to people who sent reprint requests, we also had to pay for reprints and postage. The charges for reprints were even higher for prints in color. Compared to these charges, the open-access fees are actually not that much higher in many cases, and particularly for IJWD, which is $800 for a research paper or review, $500 for a case report (the same as for JAAD Case Reports), and the fees are waived if the submission comes from a World Bank poor country. Compared to a $3,000 charge from the average predatory publisher, this is very reasonable.

However, it is not just predatory journals that we need to be aware of. Now, many bogus textbook publishers and conference organizations email doctors with invitations to speak at their meetings. There are no covered expenses but there are charges for online access to book chapters with no royalty fees to the authors. For some doctors who rarely or never are invited to speak at society meetings, these invitations are a temptation and can appear to boost their CVs. I do not know who is paying to attend these conferences, but some doctors and scientists must be; these businesses must be making profits from the conference registrations and exhibitors, or they would not keep organizing these meetings.

All of these activities dilute the reputations of decent publishers and conference organizations because it becomes more difficult to gain educational support for the main specialist society congresses. Members of the public do not realize how the vast majority of medical speakers at meetings do not receive honoraria and in fact, speaker honoraria from pharmaceutical companies and travel expense reimbursement are prohibited for speakers at some meetings, such as the American Academy of Dermatology and American Dermatologic Association. In Australia, unlike Europe, it is rare to receive pharmaceutical support to attend meetings, in part because travel is so expensive but also because of new regulations. This is good idea because only those experts who actually work in and teach other specialists in the field can be sponsored to attend meetings rather than those who just want to attend.

Jeffrey Beall’s excellent opinion piece in this issue (Beall, 2016) about these predatory medical journals should be mandatory reading for medical students and junior doctors so they are educated about these publishers and can avoid being sucked in. Likewise, those of us who are relatively new to open-access publishing need to take a more educated and broad-minded view about the benefits to society of the peer-reviewed open-access model. Not only can medical subscribers and university academics access these papers for free, but now patients and other interested parties can read articles that previously would have cost them a significant amount of money to read. Research grants and departments need to become more used to the concept of open-access publication fees for *non-predatory,* legitimate peer-reviewed journals, such as the IJWD. IJWD became aware of at least one American department of dermatology whose policy does not allow its faculty to publish in open-access journals because of the open-access fees. One of our WDS members, who is a faculty member with this department, submitted a paper to IJWD, which was accepted after peer-review and revision. The paper had to be withdrawn and, having been improved thanks to our reviewers’ voluntary efforts, is now published elsewhere. Those departments forget that behind all of these legitimate journals are editors who volunteer a considerable amount of their after-hours time, and expert reviewers who volunteer their time and savings for their departments from the old days on librarians who request papers, medical illustrations, printing fees for photographs, color images, and reprint fees. A more tolerant view of open-access publications with increased awareness and vigilance about predatory journals will help us all to maintain the standards of medical publications.
